# The role of artificial intelligence in the management of trigeminal neuralgia

**DOI:** 10.3389/fsurg.2023.1310414

**Published:** 2023-11-16

**Authors:** Marco Battistelli, Alessandro Izzo, Manuela D’Ercole, Quintino Giorgio D’Alessandris, Nicola Montano

**Affiliations:** Department of Neuroscience, Neurosurgery Section, Fondazione Policlinico Universitario A. Gemelli IRCCS, Università Cattolica del Sacro Cuore, Rome, Italy

**Keywords:** trigeminal neuralgia, neurovascular conflict, neuroanatomy, microvascular decompression, artificial intelligence, magnetic resonance imaging

## Abstract

Trigeminal neuralgia (TN) is the most frequent facial pain. It is difficult to treat pharmacologically and a significant amount of patients can become drug-resistant requiring surgical intervention. From an etiologically point of view TN can be distinguished in a classic form, usually due to a neurovascular conflict, a secondary form (for example related to multiple sclerosis or a cerebello-pontine angle tumor) and an idiopathic form in which no anatomical cause is identifiable. Despite numerous efforts to treat TN, many patients experience recurrence after multiple operations. This fact reflects our incomplete understanding of TN pathogenesis. Artificial intelligence (AI) uses computer technology to develop systems for extension of human intelligence. In the last few years, it has been a widespread of AI in different areas of medicine to implement diagnostic accuracy, treatment selection and even drug production. The aim of this mini-review is to provide an up to date of the state-of-art of AI applications in TN diagnosis and management.

## Introduction

Trigeminal neuralgia (TN) is a form of neuropathic facial pain which significantly impacts quality-of-life of affected patients (1). Typical TN presents as relapse-remitting pain, whereas in atypical TN a continuous component of pain is present (2). Etiologically, it can be divided in primary and secondary TN, the latter one due to cerebellopontine-angle tumors or multiple sclerosis. Primary TN is further divided in classic, due to a neurovascular conflict (NVC), and idiopathic, where a clear anatomical cause is missing. In a recent umbrella review microvascular decompression (MVD) emerged as the most effective treatment for classic TN (3). Even if there are continuous innovations in the field of TN, such as the understanding of the role of some biomarkers (4) and the use of morphometric magnetic resonance imaging (MRI) (5), it is not always easy to determine the etiology, and consequentially the appropriate treatment, in each patient. Artificial intelligence (AI) uses computer technology to develop systems for extension of human intelligence. It is emerging as a increasingly widespread tool in medicine to implement diagnostic accuracy, treatment selection and even drug production (6). The aim of this mini-review is to provide an up to date of the state-of-art of AI applications in TN management.

## Methods

**Figure 1:**
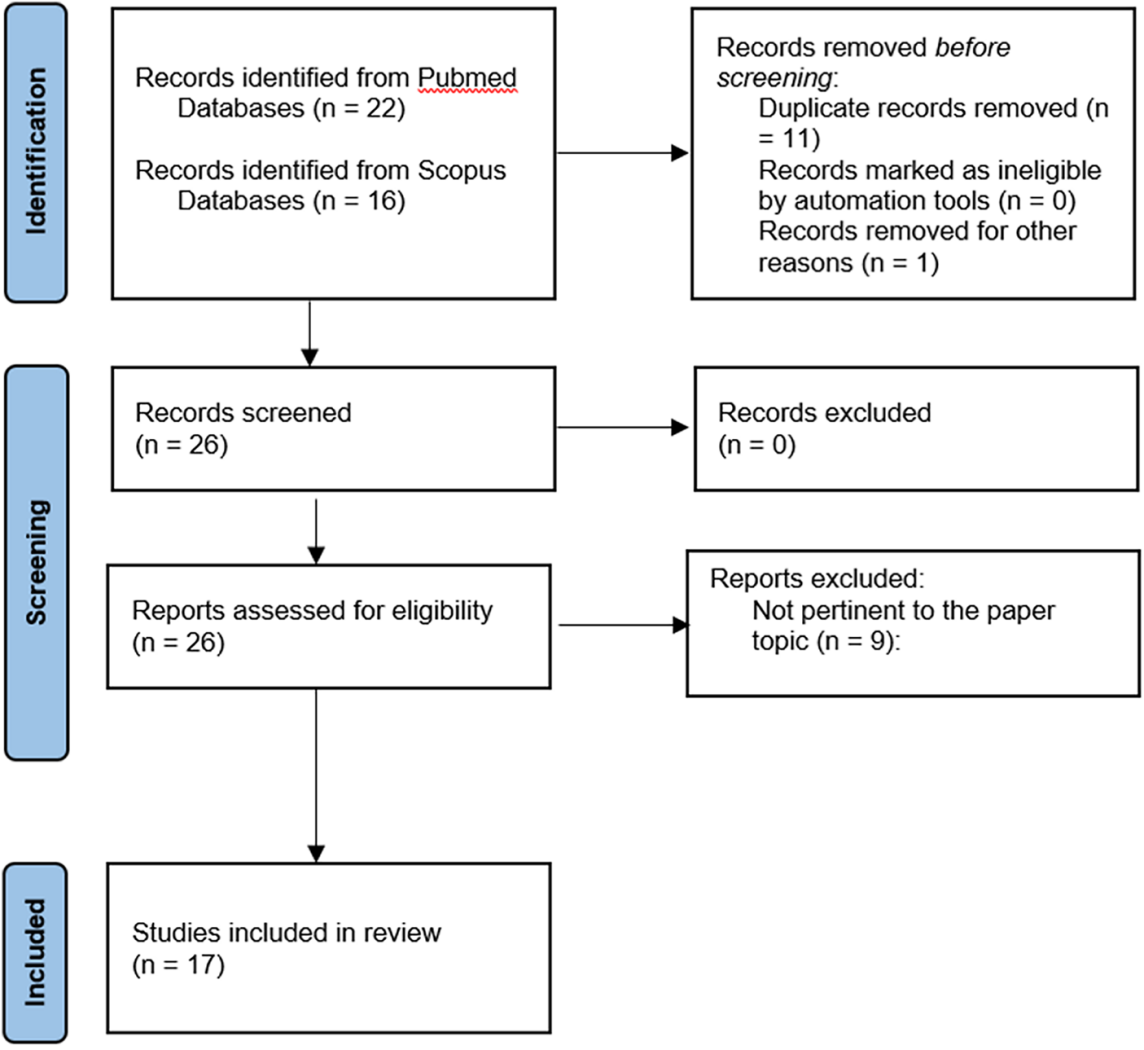
Flowchart of study selection.

Two medical databases (PubMed and Scopus) were screened for eligible scientific reports. The key words “deep learning”, “machine learning”, “artificial intelligence”, “trigeminal neuralgia”, “tic douloureux” (MeSH) have been used in any possible combination. The last search was launched in August 2023. Two reviewers (M.B., A.I.) independently screened the abstracts and the references list. Any difference was solved by consensus with a third senior author (N.M.) A total of 26 articles were identified and reviewed and finally, 17 studies were included in the present mini-review (Figure 1).

## Role of AI in the diagnosis of TN

AI has become increasingly involved in various aspects of TN diagnosis. It plays a pivotal role in identifying signature patterns within the cortical and subcortical regions, delineating the surgical anatomy of the cerebello-pontine angle, and distinguishing different types of facial pain. Liang et al. explored the capabilities of correlation and machine learning (ML) analysis methods in detecting activated cortical centers in TN patients. Interestingly, they found that certain cortical areas were only activated by conventional correlation methods, while others were detected by convolutional neural networks (CNN) and graph convolutional neural networks (GCNN). Their suggestion to combine these methods aims to achieve a more comprehensive understanding of the neural structures underlying TN pain perception (7). Zhong et al. utilized ML for the automated segmentation of white matter connectivity, comparing it with conventional analysis methods to identify alterations that could distinguish TN from healthy control subjects. Achieving a 0.96 AUC, they concluded that ML exhibits high reliability in discerning connectivity patterns between affected and non-affected groups (8). Mo et al. constructed a support vector machine (SVM) model based on T1-weighted and T2-weighted MRI data regarding cortical and subcortical morphological characteristics to diagnose TN patients. This model displayed high specificity, effectively distinguishing healthy patients from those afflicted by TN (9). Chen et al. used ML to analyze white matter fractional anisotropy differences between the affected and unaffected sides and healthy controls in both the trigeminalpontothalamic (TPT) and thalamocortical white matter (S1), achieving an 85% accuracy for TPT and 76% for S1 (10). Ge et al. used ML to extract V nerve radiomic features from 89 patients with unilateral idiopathic TN (UC-ITN), 55 of whom had NVC on the unaffected side. Small Area Low Gray Level Emphasis (SALGLE), Coarsenss, Minor Axis Length (MAL) Dependence Variance (DV), Maximum MALDV, Correlation and Offending Vessel were identified as texture features relevant to pain occurrence (11). AI also proves valuable in differentiating between various facial pain syndromes through radiographic and clinical questionnaires. Latypov et al. demonstrated 95% prediction accuracy using Random Forest (RF) supervised learning to distinguish classic TN from healthy controls based on T1-weighted and DTI MRI features. However, when attempting to differentiate between classic TN and trigeminal neuropathic pain (TNP), the accuracy dropped to 51% (12). Limonadi et al., as early as 2006, trained an Artificial Neural Network (ANN) to recognize facial pain syndrome patterns based on an 18-binomial response questionnaire. While high accuracy was achieved for classic TN assessment, the sensitivity was 50% for atypical TN and 33% for TNP (13). In a more recent study, McCartney et al. developed an ANN model based on a 22-question binomial questionnaire for distinguishing facial pain syndromes. This approach yielded high sensitivity and specificity for classic TN (92.5% and 87.8%, respectively). Remarkably, unlike Limonadi et al., they achieved high sensitivity and specificity for TNP (86.7% and 95.2%, respectively) (14). AI is also finding utility in examining peripheral V nerve characteristics. Mulford et al. employed a deep learning network to segment and extract radiomic features from the pre-ganglionic V nerve to distinguish affected from non-affected sides. Their method achieved an accuracy of 78%, specificity of 76%, sensitivity of 82%, and an AUC of 0.83, enabling reliable differentiation of TN-afflicted and pain-free nerves (15). Additionally, Lin et al. utilized an AI network to develop a V nerve and surrounding vascular structures segmentation network, aiding surgeons in planning surgery by creating a 3D model based on Magnetic Resonance Angiography (MRA). Their segmentation approach exhibited higher accuracy for V nerve segmentation (Dice similarity coefficient 0.8645, Hausdorff distance 0.2414, and average surface distance error 0.4296) compared to cerebrovascular structures segmentation (16).

## Role of AI in the treatment of TN

In contrast to Lin et al., Bai et al. introduced MVDNet, a deep learning network focused on real-time blood vessel and cranial nerve segmentation during MVD procedures for facial and trigeminal nerve disorders. MVDNet achieved impressive precision, with a 76.59% Intersection-over-Union (mIoU) accuracy and a rapid 137.6 fps speed, surpassing other real-time models (17). AI has brought about predictive models for postoperative outcomes. Hao et al. developed an ANN which forecasted long-term Barrow Neurological Institute (BNI) Scores after MVD with an accuracy rate of 95.2% and area under the curve of 0.862 (18). Goyal et al. evaluated an ANN model, trained on 16 variables, to predict post-operative outcomes following Gamma Knife Radiosurgery (GKRS). The ANN exhibited 90.9% accuracy in predicting treatment responses (19). Ertiaei et al. created a multidimensional ANN model for post-GKRS predictions, including pain reduction and hypoesthesia, achieving accuracy rates of 91.5% and 76.8%, respectively (20). Hung et al. used SVM and sequential backward selection (SBS) models with MRI data on cortical thickness and regional surface area. These models showed predictive capabilities for one-year GKRS responses, with regional surface area at 96.7% accuracy and regional cortical thickness at 90.5% accuracy (21). The same team used Gaussian Process Regression (GPR) on T1-weighted MRI data to assess brain-predicted age (Brain-AGE) differences between TN patients and healthy controls, finding a significant correlation with radiosurgery response (21). Willsey et al. employed an SVM model to predict TN recurrence post-MVD surgery, considering factors like normalized radial diffusivity (PRD) and symptom duration. The SVM model reached an 85% accuracy, 83% sensitivity, and 86% specificity (22).

## Discussion

TN affects about 15,000 people per year in the US (23). Classic TN is the most common type with with MVD as a specific etiological treatment (24). NVC identification is crucial for surgical success, but it's not always evident pre-surgery. A recent study by Jani et al. identified neurovascular compression in 18 out of 27 patients on T2-weighted FIESTA thin-cut sequence performed with a 3 T scanner (25). However, Deep et al. reported up to 53% NCV in asymptomatic patients using high-resolution MRI on 200 examined nerves (26). Different pathophysiological theories such as bioresonance hypothesis and ignition hypothesis have been developed to explain the TN pathogenesis (27). Given the crucial role of identifying NVC before MVD procedures, Lin et al. introduced trigeminal nerve segmentation method from MRA based on 3D convolutional neural network using CS2Net for tubular structure segmentation (23). The proposed method outperformed other models in segmenting the trigeminal nerve and surrounding vascular structures near the REZ, enabling accurate NVC identification, severity assessment, and surgical simulation. This is enhanced by 3D rendering, enabling direct visualization of NVC from various perspectives and zoom levels. With advancements in neuronavigation reducing bone flaps during MVD (28), AI intraoperative segmentation protocols, like Bai et al. innovative encoder-decoder structure, become invaluable for identifying vessels and nerves in tight surgical spaces (17). It is worth noting the protocol's impressive speed and accuracy, providing real-time assistance to surgeons during procedures. This was made possible by an extensive dataset comprising 3,087 MVD images labeled by experts, with 1,806 used for training. However, it is important to note that the protocol's accuracy decreased notably when applied to elderly patients (age 40–50 PICA mIoU: 74.08% vs. age 60–70: 70.33%; age 40–50 AICA mIoU: 71.43% vs. age 60–70: 68.43%), likely due to a tortuous anatomy of intracranial vessels. Limonadi et al. and McCartney et al., despite focusing on different aspects of TN management, also observed reduced accuracy in their ANN predictive models when assessing rarer pathologies [sensitivity for TN type 2 and TNP was 50% and 33%, respectively (13); sensitivity for TN type 2 and TDP was 62.5% and 0%, respectively (14)]. These reports raise concerns about AI's precision with non-linear and limited datasets, emphasizing its limited ability to draw reliable conclusions. Limonadi et al. observed improved diagnostic performance in the second set of patients, as evidenced by decreasing mean square errors during network simulations, highlighting the importance of robust training and ample data for AI model effectiveness. However, sensitivity in TN type 2 diagnosis remained unsatisfactory in the second set. The Authors attributed this to initial symptom onset resembling TN type 1, evolving into the continuous pain typical of TN type 2. This underscores the importance of initial input from experienced clinicians to determine essential diagnostic variables, as also emphasized by Mulford et al. (10) and Chen et al. (15). While ML accuracy improves with repeated calculations, manual data input can introduce bias into dataset analyses. AI can be valuable in preoperative outcome assessment and patient selection. Hao et al. identified four factors affecting ANN model performance in long-term MVD prognostication: correspondence of the neurovascular offending site with facial pain region, immediate postoperative pain remission, degree of nerve compression by culprit vessels, and culprit vessel type, while age seems to not affect long-term outcome. Recent meta-analyses highlighted predictors like isolated venous conflict (*p* < 0.01), absence of immediate postoperative pain remission (29), arterial conflict (*p* < 0.01) (30), symptom duration less than 5 years, SCA involvement, and paroxysmal pain (31). A prospective non-randomized trial documented severity of NVC (grade 2–3) as a positive predictor (*p* = 0.003) (32) and a retrospective study documented the non-inferiority of MVD in elderly people (33). Regarding GKRS long-term outcome prediction, ANN models and conventional analyses evaluated similar factors, including prior treatment, involved dermatomes, post-GKRS numbness, pain type, radiosurgery dosage, and age (19, 20, 34, 35). In summary, AI's contribution is not significantly superior to conventional statistical analyses. However, its primary advantage in routine clinical use lies in enhancing accuracy and providing real-time assistance during outpatient assessments, compensating for the limitations of empirical judgment derived from traditional statistical analyses. AI's role in TN management extends to research. Ge et al. found that 61.80% of patients analyzed had NVC on the unaffected V nerve, highlighting predictive radiomics features for symptomatic TN (11). Chen et al. observed bilateral radial diffusivity changes, even on the unaffected side of unilateral TN, differing from healthy controls (10). Microscopic degeneration in TN patients may affect not only the affected nerve but also the contralateral side, potentially contributing to chronic pain and warranting further research. While AI exhibits remarkable performance in aiding clinical practice, its seamless integration poses significant challenges. Ethical concerns, encompassing data privacy, informed consent, and patient autonomy, must be addressed. Despite its potential drawbacks, AI is an integral part of modern healthcare. Collaborative efforts between governments and clinicians are essential to establish robust regulations. In conclusion, this review highlights AI's diverse applications in TN management, showcasing its pivotal role in precise diagnosis, individualized treatment, and advancing our understanding of the condition's pathophysiology. The future holds promise for AI-driven research and enhanced patient care.
